# The Longevity of Hippocampus-Dependent Memory Is Orchestrated by the Locus Coeruleus-Noradrenergic System

**DOI:** 10.1155/2017/2727602

**Published:** 2017-06-11

**Authors:** Niels Hansen

**Affiliations:** Department of Psychiatry, University of Bonn, Sigmund Freud Str. 25, 53127 Bonn, Germany

## Abstract

The locus coeruleus is connected to the dorsal hippocampus via strong fiber projections. It becomes activated after arousal and novelty, whereupon noradrenaline is released in the hippocampus. Noradrenaline from the locus coeruleus is involved in modulating the encoding, consolidation, retrieval, and reversal of hippocampus-based memory. Memory storage can be modified by the activation of the locus coeruleus and subsequent facilitation of hippocampal long-term plasticity in the forms of long-term depression and long-term potentiation. Recent evidence indicates that noradrenaline and dopamine are coreleased in the hippocampus from locus coeruleus terminals, thus fostering neuromodulation of long-term synaptic plasticity and memory. Noradrenaline is an inductor of epigenetic modifications regulating transcriptional control of synaptic long-term plasticity to gate the endurance of memory storage. In conclusion, locus coeruleus activation primes the persistence of hippocampus-based long-term memory.

## 1. Introduction

The locus coeruleus (LC) resides in the brainstem's dorsal pons, is the main origin of noradrenaline (NA) in the central nervous system, and is linked to the hippocampus [1], thus being essential for hippocampus-based declarative memory formation [2]. Nevertheless, LC projections are ubiquitous in the brain, targeting other brain structures involved in memory formation such as the amygdala [3] and the prefrontal cortex [4]. However, its projection specificity encompasses unique roles in memory processes [5]. The LC-NA system regulating memory function must be considered as an orchestra composed of different neural circuits that are functionally linked to the hippocampus, such as the amygdala [6] or prefrontal cortex [2] receiving projections from the LC [3, 4] thus making them subject to NA modulation. The orchestra's function is guaranteed by each neuronal circuit's activity.

## 2. Noradrenaline Release after Locus Coeruleus Activation

**Figure 1:**
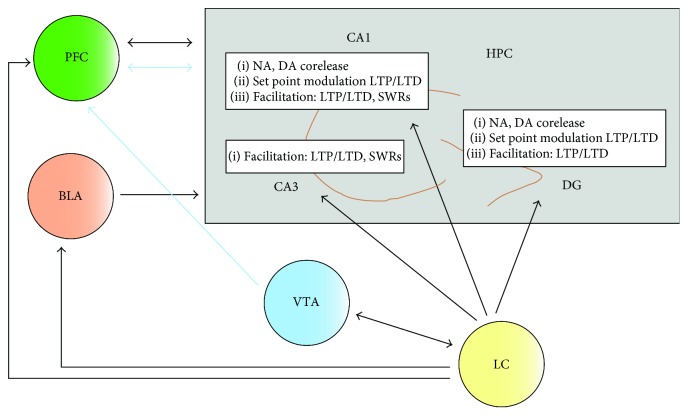
Priming of hippocampus-based memory via locus coeruleus activation. The ventral tegmental area (VTA) and LC are interlinked by fiber projections [35]. After locus coeruleus (LC) activation, noradrenaline (NA) and dopamine (DA) are released in the dentate gyrus of the hippocampus from LC terminals [11, 13]. The LC projects also to the CA1 and CA3 region of the hippocampus [82]. The main mechanisms involved in how memory is primed by NA and DA are indicated in boxes at specific hippocampal subregions [25–28, 33, 63, 72]. Moreover, two other brain structures such as the basolateral amygdala (BLA) and the prefrontal cortex (PFC) receive projections from the LC [3, 4] and participate in noradrenergic and dopaminergic modulation of hippocampus-based memory [6, 49, 61]. BLA = basolateral amygdala, DG = dentate gyrus, HPC = hippocampus, LC = locus coeruleus, LTP = long-term potentiation, LTD = long-term depression, PFC = prefrontal cortex, SWRs = sharp wave ripples, VTA = ventral tegmental area.

The LC is activated after novelty [7] and arousal [8]. NA is released within the LC after its activation [9, 10]. In addition, electrical activation of the LC leads to NA release in the rodent dentate gyrus [11], an important input structure in the hippocampus (Figure 1). A model of LC function proposed by Atzori et al. [12] related the NA concentration in different brain activation states regulating sleep and wakefulness with the activation of *α*1-, *α*2-, and *ß*-adrenoreceptors. *ß*-adrenoreceptors are believed to be activated by interplay between tonic and phasic firing of LC neurons [12] in the hippocampus that is innervated by LC projections [13] and richly endowed with *ß*-adrenoreceptors [14, 15].

The noradrenergic system's importance and modulatory role in forming memories was postulated by Kety in the 1970s [16, 17]. A decade later, this hypothesis was confirmed by experimental data in the rodent hippocampus. Harley's group was the first to demonstrate that applying NA can enhance the spike activity of the field potential in the dentate gyrus elicited by stimulating the perforant pathway [18] which is a major input pathway to the hippocampus connecting the entorhinal cortex with the dentate gyrus. Furthermore, NA depletion in the dentate gyrus promotes long-term potentiation (LTP) [19]. These findings suggest NA's major role in hippocampal LTP and memory, as LTP is considered a cellular mechanism of learning and memory [20].

## 3. Memory Encoding and Consolidation Are Promoted by Locus Coeruleus Activation

Early experiments in rats in the 1970s revealed that bilateral LC lesions can impair hippocampus-based spatial memory encoding assessed by the T-maze task [21] (see Table 1 for examples of memory modulation via LC activation). Memory consolidation is a key step toward building robust long-term memories. In the same decade, another group demonstrated by electrolytic LC lesions in mice that the LC is essential to this step in consolidating memory within a critical time period [22]. Experiments in rats two decades later revealed that the LC is involved in spatial and nonspatial learning processes [23], demonstrating that unilateral LC lesions lead to slightly, and bilateral LC lesions to strongly affected nonspatial and spatial memory functions [23]. Memory consolidation is further influenced by the occurrence of sharp wave ripples. These are patterns of cortical oscillations that circulate and transfer information as hippocampal representations between the entorhinal cortex and hippocampus to other brain circuits in order to enable memory consolidation. Mostly, sharp wave ripples arise from the hippocampus' CA3 subregion and originate during sleep or immobility [24]. In vitro experiments in the rat indicated that *ß*-adrenoreceptor agonism can facilitate sharp wave ripples and LTP [25], supporting the NA's role in modulating sharp wave ripples as well as synaptic plasticity and thereby hippocampal representations to consolidate memory (Figure 1).

## 4. Long-Term Synaptic Plasticity Is Modulated by Locus Coeruleus Activation

Nowadays, however, there is evidence that LC activation does not just enhance LTP in rodents [26]—it also facilitates long-term depression (LTD) [27, 28] as another putative mechanism of cellular memory storage [29] (Figure 1). High-frequency electrical stimulation of the LC combined with test pulse stimulation of input pathways to hippocampal subfields such as the (1) perforant path and (2) the Schaffer collaterals resulted in LTD in the dentate gyrus or CA1 region of rats [27, 28]. The modulation of LTP and/or LTD via LC activation highlights the LC's crucial role in selecting important information for further long-term storage. Electrophysiological and behavioral animal data indicate that LTD's supposed role in forgetting is overly simplistic. LTD also serves to encode fine spatial details in an environment as demonstrated in an in vivo study in rats showing facilitated LTD after exploring objects in new locations, whereas exploration of the novel environment without objects impaired LTD [30]. In contrast, LTP is facilitated in rats if they explore an empty holeboard as an indicator for global space [30]. Considering LTD's aforementioned roles such as encoding fine spatial details [30, 31] and of LTP—the encoding of the global environment [30, 32]—the LC's modulatory function seems to contribute to both aspects of spatial memory and relies largely on activation of *ß*-adrenoreceptors [27].

However, both *ß*-adrenoreceptors [27] and dopamine D1/5 receptors [33] are key mediators for LC-induced LTD in rodents. D 1/5 receptor agonism during novel environmental exploration promotes LTD in the CA1 region over 24 hours, whereas LC-induced LTD is blocked by a dopamine D1/5 receptor antagonism in the rat [33]. These animal study findings led to the conclusion that dopamine D1/5 receptor agonism is capable of priming late LTD depending on protein synthesis [34]. This in turn suggests that dopamine D1/5 receptors play a role in persistent memory storage. The same facilitated late LTD phenomenon was observed in the rat in perforant path-dentate gyrus synapses when a *ß*-adrenoreceptor agonist was applied prior to electrical LC activation [28]. Thus, LTD can be facilitated by both the application of a D1/5 receptor and *ß*-adrenoreceptor agonist prior to the LC activation, meaning that NA acting on *ß*-adrenoreceptors, in addition to dopamine (DA) activating D1/5 receptors are important for long-term memory storage. Moreover, the enhancement of spatial memory episode is critically dependent on the *ß*-adrenoreceptors after LC activation, as demonstrated in an episodic-like memory task [27].

## 5. Memory Consolidation Depends on the Corelease of Noradrenaline and Dopamine via Locus Coeruleus Terminals in the Hippocampus

The LC is reciprocally interlinked with the ventral tegmental area (VTA) [35] (Figure 1). Furthermore, other immunohistochemical studies support the direct connection from the VTA to the LC [36, 37]. The interaction of these brainstem structures is highly relevant for the modulation of synaptic long-term plasticity and memory, as DA deriving from the VTA might be released from LC terminals in the hippocampus [13] modulating synaptic plasticity and memory via D1/5 receptor activation [38] (Figure 1). Recent evidence indicates that the LC and VTA control the synthesis of plasticity-related proteins (PRPs) for a synaptic tag [39] to promote the storage and consolidation of a memory at the site where the synaptic tag was initiated. Viral-tracing experiments revealed prominent LC and very few VTA fibers projecting into the dorsal part of hippocampus in rodents [40]. Further retrograde tracing techniques exhibited cells with retrograde labels only in the LC, not in the VTA, indicating that the LC and not the VTA sends functionally relevant projections to the hippocampus. Optogenetic and electrophysiological animal studies confirmed the LC's function in amplifying LTP via a dopamine D1/D5 receptor and not *ß*-adrenoreceptor-dependent mechanism [40]. Further immunohistochemical studies proved DA's release from the LC into the dorsal hippocampus. In addition, optogenetic activation of noradrenergic LC neurons in rodents led to an enhancement of spatial memory that was dependent on D1/5 receptors, but not *ß*-adrenoreceptors [41]. These findings seem to imply that memory consolidation is enhanced by the corelease of NA and DA in the dorsal hippocampus [40, 41] through the LC to hippocampus pathway (Figure 1). DA's role in memory encoding is not yet fully understood, but there is recent evidence that it might help encode memory by diminishing stimuli perception that interferes with memory formation [42] and by making stimuli salient for subsequent memory encoding [38].

DA and NA seem to modulate memory formation in complementary fashion. The conditions resulting in a NA and DA release differ substantially. LC neurons are activated after novelty [7], arousal [8], and aversive or reward-related stimuli as well [43, 44]. However, VTA neurons also respond to novelty, arousal, and aversive or reward-related stimuli [45–48]. Which of these conditions leads preferentially to the activation of the LC or VTA neurons remains an open question. The different release conditions of NA and DA may indicate that the two occupy different facets in memory function. A study in rats revealed such different NA and DA effects on memory with several opposite effects. Both the antagonism of dopamine D1/5 receptors and the agonism of *ß*-adrenoreceptors in the hippocampus impaired social recognition memory in rats [49].

## 6. Impact of the Amygdala on the Noradrenergic and Dopaminergic Modulation of Hippocampus-Dependent Memory

Social recognition memory depends on the interaction between the hippocampus and basolateral amydala [49]. Coinfusion of a dopamine D1/5 receptor antagonist in combination with a *ß*-adrenoreceptor agonist in the CA1 region and a dopamine D1/5 receptor agonist together with a *ß*-adrenoreceptor antagonist in the basolateral amygdala impede social recognition memory [49]. These findings indicate that social recognition memory is controlled by both dopamine D1/5 receptors and *ß*-adrenoreceptors in the CA1 region of the hippocampus and basolateral amygdala. The latter is involved not only in social recognition but also in hippocampus-based and prefrontal cortex-dependent memory [6] as proven indirectly by a recent in vivo study in rats showing that the basolateral amygdala can regulate hippocampal-prefrontal cortex LTP via alpha_2_- and *ß*-adrenoceptors [6] as a possible memory-storage mechanism. These animal data may lead me to presume that there is an NA-dependent neuronal pathway between the amygdala, hippocampus, and prefrontal cortex starting with LC projections to the basolateral amygdala [3] (Figure 1). In addition, these experimental data might suggest that the basolateral amygdala is critically involved in the noradrenergic and dopaminergic modulation of hippocampus-dependent memory.

## 7. Memory Retrieval and Reversal Are Triggered by Locus Coeruleus Activation

Memories are both stored and more rapidly retrieved in conjunction with LC activation [50]. The facilitation of memory retrieval by NA was confirmed in two further experimental studies [51, 52]. The increase in NA in one of those studies resulted from the blockade of *α*2-adrenoreceptors [51]. This is likely related to the increased firing rate of LC neurons with consecutive NA release in the hippocampus due to an antagonism of the *α*2-adrenoreceptor's inhibitory receptor properties [53] (Table 1). In the other study, LC stimulation caused a facilitated memory retrieval that was blocked by pretreatment with a *ß*-adrenoreceptor antagonist [52] (Table 1). In conclusion, the promoted memory retrieval in both studies was probably mediated by activating *ß*-adrenoreceptors.

Memory formation is a dynamic process at each memory stage. Memories are often labile and can be destabilized if they are not reconsolidated after retrieval. Reconsolidation is a memory phase that is required for the persistence of a memory trace [54]. Sara proposed that dynamic memory stages such as consolidation or reconsolidation are modulated by the LC-NA system [55]. Other studies indicated that the LC-NA system also has an impact on memory reversal [56] and extinction [57] (Table 1). The NA-dependent modulation of memory stages might be influenced by interactions between NA and other neurotransmitters, for example, with glutamate that is important for synaptic excitation and long-term synaptic plasticity. It interacts locally with NA released from the LC to augment important neuronal representations and to choose among them for long-term memory storage (as recently hypothesized in the “Glutamate Amplifies Noradrenergic effects” (GANE) theory [58]).

## 8. Locus Coeruleus Modulation of Prefrontal Cortex Activity Controls Hippocampus-Based Memory

Recent evidence suggests that the prefrontal cortex is almost as important as the hippocampus for encoding memory and memory retrieval [2]. Eichenbaum proposed a circuit model of prefrontal-hippocampal interactions to support memory formation [2]. In his model, the prefrontal cortex receives contextual information via the ventral hippocampus and controls memory retrieval by projections from the prefrontal cortex to the dorsal hippocampus [2]. The LC [1] and VTA [59] are known to project to the prefrontal cortex. Memory retrieval suppression is induced through the prefrontal cortex's modulation of hippocampal activity [60] suggesting that the prefrontal cortex can modulate hippocampus-dependent memory. There is recent evidence that application of a dopamine D 1/5 receptor antagonist in the dorsal hippocampus or medial prefrontal cortex impairs object recognition memory, whereas dopamine D1/5 receptor agonism facilitates objection recognition memory in rats [61]. Moreover, the NA transporter inhibitor reboxetine also facilitates object recognition memory in these rodents [61]. This facilitated that object recognition memory can be reversed by the antagonism of D1/5 receptors in the prefrontal cortex [61]. These findings highlight the key role of the LC-induced release of NA and LC- and VTA-induced release of DA in the prefrontal cortex in modulating memory that result from interplay between the hippocampus and prefrontal cortex (Figure 1).

## 9. Memory Priming by Locus Coeruleus Activation

NA is known to induce epigenetic modifications (for instance DNA methylation, histone acethylation, and/or phosphorylation) that regulate the transcription for synaptic long-term plasticity in the murine CA1 region in vitro [62]. NA might shape the activation matrix of synapses and further response of synapses to new incoming stimuli, that is, in the murine CA1 region in vitro [63], a concept termed metaplasticity [64, 65]. Metaplasticity is a neurophysiologic phenomenon that serves to enable robust memories by selecting and filtering information via changes in synaptic plasticity. Moreover, it might result from experience-dependent changes in synaptic plasticity driven by epigenetic modifications of transcriptional genes, that is, DNA methylation [66]. Moreover, both LC activation and interaction with other drugs such as atypical antipsychotics such as clozapine and olanzapine or nicotine may promote hippocampal metaplasticity [67]. This concept of NA-induced metaplasticity might shift or reset the sliding threshold for hippocampal synaptic plasticity. By shifting the set point, the response to new incoming stimuli changes, potentially inducing modifications in synaptic long-term plasticity. On the cellular level, this set point is decisive for the resultant type of plasticity such as LTD or LTP. The set point can be considered as an adjustable threshold for inducing LTD or LTP that favors LTP or LTD. The latter are known to regulate spatial memory formation in complementary fashion [30, 31] with their unique roles in spatial memory as depicted above. It is thus tempting to postulate a shifting set point for hippocampal memory storage by LC activation and consecutive NA release in the hippocampus analogous to that for the bidirectional synaptic plasticity exemplified in the visual system [68, 69]. As derived from animal studies, this set point modulation by LC activation is believed to occur in the hippocampal CA1 region and dentate gyrus, but is not limited to those shown in Figure 1. A set point adjustment is likely in these hippocampal subfields, as the LC's activation facilitates LTD in these regions (to test pulses that per se do not evoke changes in basal synaptic transmission) [27]. However, how exactly the amount and duration of NA and/or DA release after LC activation alters the set point for memory storage remains an open question. Here, the timing of LC activation seems to be decisive [26]. For example, activating the LC before the high-frequency stimulation (HFS) of perforant path input fibers to the dentate gryrus inhibited short-term potentiation, whereas the same LC activation after applying HFS depotentiated LTP in rats [26]. These findings lead me to presume that the timing of LC activation is crucial for the persistence of a memory trace. Whether LC reactivation reoccurs minutes after a novel or salient stimulus that per se activates the LC immediately after novel stimuli begin [70] appears to be highly relevant for the encoding of those novel or salient stimuli into long-term memory. Identifying these temporal activation characteristics could prove to be a key step in discovering how NA gates memory priming. My assumption is that the amount of NA release at each time due to LC activation is what regulates the set point for memory modulation. I base this assumption on experiments showing that hippocampal LTD and LTP in the dentate gyrus is dependent on the *ß*-adrenoreceptor agonist concentration in the rat. Lower concentrations of *ß*-adrenoreceptor agonist elicit LTD, whereas higher concentrations of the *ß*-adrenoreceptor agonist cause LTP [71], suggesting that a higher hippocampal NA concentration (resulting from a phasic or high tonic LC activation and a lower hippocampal NA concentration after a low tonic LC activation) might shift the set point for LTD/LTP induction.

Another intriguing candidate for a set point modulation triggered by LC activation is cortical oscillations. We know for one that LC activation is followed by an increase in theta power parallel to the LTP in rodents [72]. On the other hand, no LTP was observed when gamma frequencies are ameliorated after LC activation [72]. LC-facilitated CA1 LTD in rats is accompanied by the transient suppression of theta frequencies [27], which suggests that a theta frequency increase or suppression after LC activation might be responsible for directing synaptic plasticity (LTP or LTD) and forming subsequent memories. Although the precise mechanisms of set point modulation remain unclear, there are several factors that argue for the presumption that the LC primes hippocampal memory.

## 10. Concluding Remarks and Implications

Considered together, the LC-NA system comprises an essential function in modulating the stages and persistence of hippocampus-dependent memory. In several human disease states involving LC impairment, LC neurons are lost, such as in Alzheimer's disease [73] and in posttraumatic stress disorder, NA's availability is reduced [74]. In temporal lobe epilepsy, hippocampal neurons are often lost due to hippocampal sclerosis with consecutive suspected altered noradrenergic function based on LC projections to the hippocampus.

LC dysfunction thus contributes to the underlying pathophysiology of these diseases, knowledge that could help us identify factors that protect the LC from degeneration and to identify patients in an early state of Alzheimer's disease [73]. In a recent study, patients with amnestic mild cognitive impairment exhibited a 30% loss of neuronal cells in the LC [75]. Those patients may have a prodromal stage of Alzheimer's disease. In patients clinically diagnosed with Alzheimer's, LC neuronal loss was further enhanced, as detected in the patients with amnestic mild cognitive impairment [75], suggesting a progressive loss of neurons in the LC characteristic of the neurodegenerative process and believed to correlate with cognitive dysfunction. LC neurodegeneration's molecular pathology was analyzed in tissue samples from deceased patients with amnestic mild cognitive impairment, revealing reductions in messenger ribonucleic acids in synaptic structural plasticity [75] believed to be important for memory storage [76], highlighting the important role that the loss of noradrenergic LC cells plays in the development of cognitive dysfunction in Alzheimer's disease. There is ongoing debate as to which drugs might be theoretically preferable for patients with Alzheimer's disease: adrenergic drug blockage or adrenergic drug stimulation [77]. The debate is based on experimental data in Alzheimer animal models. Adrenergic drug blockage has been observed to alleviate cognitive deficits and the neuropathological changes in Alzheimer's disease such as amyloid beta and tau pathology [78]. On the other hand, adrenergic receptor activation might promote neurogenesis [79] and reduce neuroinflammation and amyloid beta and tau pathology [80].

In another disease affected by LC dysfunction, namely, posttraumatic stress disorder, the reduced availability of noradrenaline transporter is the basic idea behind developing NA reuptake blockers that cause anxiolytic effects in anxious arousal states [74]. Moreover, in an animal model of focal hippocampal epilepsy, electrical LC stimulation via activation of *ß*-adrenoreceptors reduced hippocampal epileptic activity [81].

It is therefore important that we understand LC pathophysiology in these disease states so as to design drugs to help restore LC dysfunction.

To sum up, I propose that the cellular plasticity mechanisms induced by LC activation listed below are among the mechanisms that regulate the persistence of long-term memory (Figure 1):

- Facilitation of synaptic hippocampal LTD and/or LTP via the corelease of NA and DA in the hippocampus [26–28, 33]. In particular, the noradrenergic and dopaminergic modulation of late LTD facilitated by electrical LC activation is of major relevance in the formation of long-term memory (Figure 1).
- Facilitation of hippocampal sharp waves ripples via *ß*-adrenoreceptors after NA release in the hippocampus (Figure 1). This mechanism was proven in an in vitro study in the rodent [25]. This study implies an improvement in memory consolidation via increased hippocampal sharp wave ripples.
- NA-induced epigenetic modifications of transcriptional control of synaptic hippocampal long-term plasticity. This proposed mechanism was demonstrated in an in vitro study in the CA1 region [63].
- NA-elicited shifts of the set point for LTP and/or LTD (Figure 1) causing hippocampal metaplasticity. This is a hypothetical mechanism demonstrated indirectly in experiments. NA is shown on the one hand to facilitate LTD and thus to lower the threshold for inducing LTD in hippocampal synapses. On the other hand, the LTP threshold is modulated via NA as LTP and is depotentiated when LC activation follows immediately after LTP induction [26]. It is thus reasonable to assume that an LC-induced NA release shifts the thresholds inducing hippocampal long-term plasticity. However, the exact molecular mechanism by which NA sets the threshold of synaptic long-term plasticity remains unclear. On the network level, potential mechanism candidates for the threshold shifting of LTP or LTD are an NA-facilitated increase or suppression in theta frequencies [27, 72]. It is conceivable that the set point modulation is also induced by DA released from LC terminals.

Taken together, these mechanisms based on the reviewed literature lead me to assume that the LC-NA system's pivotal role is to prime the longevity of hippocampal long-term memory.

## Figures and Tables

**Table 1:** Modulation of hippocampus-dependent memory via locus coeruleus activation.

Memory stages	Method of LC activation/suppression	Effect on memory	Reference
*Encoding*	Bilateral LC lesions	Impaired spatial memory in T-maze	[21]
	Electrical LC stimulation with 100 Hz	Improved acquisition of food-reinforced task	[57]
	Bilateral/unilateral LC lesions	Unilateral mildly, bilateral severely impaired memory assessed by Greek cross version of water maze	[23]
	LC clonidine injection	Deficits in attention, radial maze: no effect on working memory	[83]
	Electrical LC stimulation with 100 Hz	Promoted encoding of spatial memory via *ß*-adrenoreceptor activation	[27]
	LC lidocaine injection	Impaired acquisition of reference and working memory	[84]
	DSP 4 treatment in APP/PS1 mice	Exacerbation of short-term olfactory memory deficits	[85]
	Immunotoxic ablation of LC neurons	Water maze task: working memory deficits	[86]
	Photostimulation of LC axons	Spatial object recognition memory enhancement, D1/5 receptor dependent	[41]

*Consolidation*	Electrolytic LC lesions	Memory consolidation is achieved	[22]
	LC lidocaine injection	Affected memory retention in an inhibitory avoidance task after training impaired memory consolidation	[87]
	LC muscimol microinfusion	Impaired object recognition memory consolidation	[88]
	Electrical LC stimulation with 100 Hz	Caused reference memory deficit	[89]
	Electrical LC stimulation with 20 Hz	No effect on spatial learning	[89]
	Photostimulation of LC TH+ neurons	Novelty associated memory enhancement, D1/5 receptor dependent	[40]

*Retrieval*	Electrical LC stimulation	Facilitated memory retrieval	[50]
	Idazoxan treatment	*α*2 receptor antagonism enhance memory retrieval	[51]
	Electrical LC stimulation	Reduced forgetting via activation of *ß*-adrenoreceptors	[52]
	LC agmatine infusion	Facilitated memory retrieval, yohimbine facilitated, whereas clonidine attenuated the effects of agmatine within the LC	[90]

*Extinction*	Electrical LC stimulation with 100 Hz	Improved extinction of food-reinforced task	[57]

APP/PS1: amyloid precursor protein/presenilin 1; D1/5: dopamine D1/5 receptors: Hz: hertz; LC: locus coeruleus; min: minutes; SP4: N-(2-chloroethyl)-N-ethyl-bromo-benzylamine; LC: locus coeruleus; TH+: thyrosine hydroxylase positive.
